# Tailored Polymer-Based Selective Extraction of Lipid Mediators from Biological Samples

**DOI:** 10.3390/metabo11080539

**Published:** 2021-08-13

**Authors:** Yohannes Abere Ambaw, Sandra Rinne Dahl, Yan Chen, Tyge Greibrokk, Elsa Lundanes, Issam Lazraq, Sudhirkumar Shinde, Jayashree Selvalatchmanan, Markus R. Wenk, Börje Sellergren, Federico Torta

**Affiliations:** 1 Precision Medicine Translational Research Programme and Department of Biochemistry, Yong Loo Lin School of Medicine, National University of Singapore, Singapore 119077, Singapore; yambaw@hsph.harvard.edu (Y.A.A.); jayashree.s@u.nus.edu (J.S.); bchmrw@nus.edu.sg (M.R.W.); 2 SLING, Singapore Lipidomics Incubator, Life Sciences Institute, National University of Singapore, Singapore 119077, Singapore; 3 Department of Molecular Metabolism, Harvard T.H. Chan School of Public Health, Harvard University, Cambridge, MA 02138, USA; 4 Department of Chemistry, University of Oslo, 0315 Oslo, Norway; sadahl@ous-hf.no (S.R.D.); notavailable@gmail.com (Y.C.); tyge.greibrokk@kjemi.uio.no (T.G.); elsa.lundanes@kjemi.uio.no (E.L.); 5 Hormone Laboratory, Department of Medical Biochemistry, Oslo University Hospital, 0424 Oslo, Norway; 6 Department of Biomedical Sciences, Biofilms Research Center for Biointerfaces, Faculty of Health and Society, Malmö University, 21119 Malmö, Sweden; ilazraq@dow.com (I.L.); sudhirkumar.shinde@mitwpu.edu.in (S.S.); 7 School of Consciousness, Dr Vishwanath Karad Maharashtra Institute of Technology–World Peace University, Kothrud, Pune 411038, Maharashtra, India

**Keywords:** lipid mediators, molecularly imprinted polymer (MIP), non-imprinted polymer (NIP), strata-X, solid-phase extraction (SPE)

## Abstract

Lipid mediators, small molecules involved in regulating inflammation and its resolution, are a class of lipids of wide interest as their levels in blood and tissues may be used to monitor health and disease states or the effect of new treatments. These molecules are present at low levels in biological samples, and an enrichment step is often needed for their detection. We describe a rapid and selective method that uses new low-cost molecularly imprinted (MIP) and non-imprinted (NIP) polymeric sorbents for the extraction of lipid mediators from plasma and tissue samples. The extraction process was carried out in solid-phase extraction (SPE) cartridges, manually packed with the sorbents. After extraction, lipid mediators were quantified by liquid chromatography–tandem mass spectrometry (LC–MSMS). Various parameters affecting the extraction efficiency were evaluated to achieve optimal recovery and to reduce non-specific interactions. Preliminary tests showed that MIPs, designed using the prostaglandin biosynthetic precursor arachidonic acid, could effectively enrich prostaglandins and structurally related molecules. However, for other lipid mediators, MIP and NIP displayed comparable recoveries. Under optimized conditions, the recoveries of synthetic standards ranged from 62% to 100%. This new extraction method was applied to the determination of the lipid mediators concentration in human plasma and mouse tissues and compared to other methods based on commercially available cartridges. In general, the methods showed comparable performances. In terms of structural specificity, our newly synthesized materials accomplished better retention of prostaglandins (PGs), hydroxydocosahexaenoic acid (HDoHE), HEPE, hydroxyeicosatetraenoic acids (HETE), hydroxyeicosatrienoic acid (HETrE), and polyunsaturated fatty acid (PUFA) compounds, while the commercially available Strata-X showed a higher recovery for dihydroxyeicosatetraenoic acid (diHETrEs). In summary, our results suggest that this new material can be successfully implemented for the extraction of lipid mediators from biological samples.

## 1. Introduction

Lipid mediators are low abundant signaling molecules mainly originated from arachidonic acid (ArA), eicosapentaenoic acid (EPA), or docosahexaenoic acid (DHA) under the catalysis of cyclooxygenase (COX), lipoxygenase (LOX), or cytochrome P450 (CYP). According to their precursors, lipid mediators can be divided into two categories, omega-6 (ω-6) and omega-3 (ω-3). The ω-6 lipid mediators are mainly derived from ArA and include 2-series prostaglandins (PG), thromboxanes (TX), 4-series leukotrienes (LT), lipoxins (LX), hydroxyeicosatetraenoic acids (HETE), epoxyeicosatrienoic acids (EET), and their corresponding dihydroxyeicosatrienoic acid (DHET) products [1]. When considering ω-3 lipid mediators, 3-series PGs and TXs, 5-series LTs and LXs, hydroxyeicosapentaenoic acid (HEPE), epoxyeicosatetraenoic acid (EpETE), and dihydroxyeicosatetraenoic acid (diHETE) are derived from EPA, while hydroxydocosahexaenoic acid (HDoHE), epoxydocosapentaenoic acid (EpDPA), and dihydroxydocosapentaenoic acid (DiHDPA) are derived from DHA [2]. The biological role of many lipid mediators has not been fully clarified yet; however, several of them have been implicated in the regulation of physiological processes such as blood pressure [3], inflammation [4], pain [5], and cellular proliferation [4]. A quantitative analysis of lipid mediators in biological samples is the key to a mechanistic understanding of their biological roles and is usually achieved by liquid chromatography–mass spectrometry (LC–MSMS) [6,7].

Other than the analytical method for their detection, it has been shown that sample extraction methods considerably influence the quantitation of these metabolites. Removal of proteins and other contaminating molecules is crucial to increase the sensitivity of the detection of lipid mediators. The simplest method to extract lipid mediators from biological samples involves the addition of acidified aqueous and organic solvents. However, this method can be complicated by the formation of emulsions, reducing the efficiency of the extraction. To improve the process, methods based on solid-phase extraction using columns or plates were developed [8,9,10]. This procedure brought several advantages, as it is faster, it removes many contaminants, and the extract is obtained in a small solvent volume that can be readily evaporated before mass spectrometry-based analysis. This technique can also be adapted for the extraction of large amounts of the initial sample, as high capacity SPE devices can be used [11]. Different solid-phase extraction (SPE) protocols have been developed and described [7,12]. In the most popular applications, commercially available polymeric stationary phases containing polar groups, such as Oasis HLB (Waters, Eschborn, Germany) [12] or Strata-X (Phenomenex, Torrance, CA, USA) [7], are usually employed. However, the cost of these devices might be limiting for laboratories that have high-throughput requirements. Therefore, we explored the synthesis of alternative polymeric materials as a more affordable solution for the selective extraction of lipid mediators [13,14,15,16]. 

Molecularly imprinted polymers (MIPs), stable synthetic polymers possessing selective molecular recognition sites, are obtained by crosslinking monomers in the presence of the target molecule (template) [16]. After polymerization, the template is removed, leaving specific cavities that can selectively re-bind the same target molecules (such as lipid mediators) based on size, shape, and functionality. The advantages of MIPs, such as stability at extreme values of pH and temperature, ease of preparation, low cost, and reusability, have led to the development of various MIP applications for chromatographic separations, chemical sensors, and catalysis [13,14,15,16].

One of the most useful applications of MIPs is their use as a sorbent in SPE [17,18]. Here, the sample is passed through a cartridge (or a packed column) filled with a solid sorbent to which the analytes bind and are released in the presence of specific solvents (Figure S1). The SPE-based procedure can present several advantages over a liquid–liquid extraction (LLE): it is faster, decreases the use of toxic solvents and facilitates automation, offering the possibility to enrich low abundant compounds [19,20]. 

Especially for low abundant compounds such as lipid mediators and before LC–MS quantification, the SPE-based extraction is necessary [7,21,22] to avoid the time-consuming and laborious steps of hydrolysis and derivatization that may introduce a higher variability [23]. The SPE step is also necessary to remove interfering matrix components during the concentration of other low abundance analytes, such as PGs. However, this also increases the cost of the process, as cartridges can be expensive and, most of the time, are single-use only. As an alternative to this well-established methodology for the enrichment of lipid mediators, we considered the use of molecularly imprinted sorbents, which combine an effective cleanup and highly selective enrichment with high ruggedness and good compatibility with online LC [24,25].

In the present study, we assessed new arachidonic acid-imprinted polymers for their ability to bind lipid mediators and compared their performances with both non-imprinted polymers and the commercially available and widely used Strata-X [26] for the analysis of human plasma and murine tissues. The advantages associated with the use of these new materials are cost-effectiveness, higher stability to experimental conditions, longer shelf life, reusability, specificity, and selectivity for target analytes [27].

## 2. Results and Discussion

### 2.1. MIP Synthesis and Characterization

Molecular imprinting entails co-polymerization of mono- and poly-functional monomers in the presence of a template, which represents the molecule of interest to be isolated from complex samples, that is thereafter removed to leave sites that can be re-occupied by the template or closely related compounds when in use. This leaves a highly crosslinked and durable molecularly imprinted polymer (MIP) containing templated sites or imprints of the template added in the polymerization step. 

The most versatile approach to the synthesis of molecularly imprinted sorbents is based on self-assembly of the template and a complementary functional monomer prior to polymerization. Thus, the template remains associated with the growing polymer during synthesis and, by adding a large portion of a crosslinking monomer (e.g., ethylenglycoldimethacrylate (EGDMA)), sites complementary to the template are formed and remain stable after template removal. The use of solvents of low polarity and the addition of an excess of functional monomer are the common means ensuring that the template molecule is complexed to a maximal degree.

**Figure 1:**
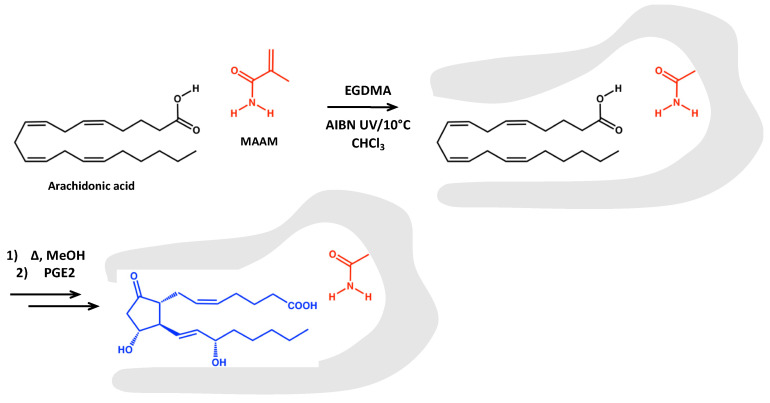
Dummy template imprinting scheme to produce MIPs complementary to prostaglandins. The initial template molecule (arachidonic acid) is represented in black, while the analyte to be bound (PGE2) in the samples is represented in blue.

The engineering of MIPs, capable of recognizing a group of structurally related compounds, is a key activity in MIP development. Hence, the use of “dummy” templates has been exploited to achieve class-selective MIPs for a broad range of compound classes. Synthesis of MIPs complementary to lipid mediators, such as prostaglandins, is generally difficult due to the non-availability or high costs of the template. To overcome this difficulty, we considered the biosynthetic pathway of these molecules to find commercially available precursors [28,29]. The PG precursor arachidonic acid (ArA) is readily available for this purpose. This fatty acid is soluble in its protonated form in apolar organic solvents, and we considered its compatibility with conventional non-aqueous imprinting using hydrogen bond donor–acceptor monomers such as methacrylamide (MAAM) (Figure 1). Based on the “bait and switch” principle, the resulting arachidonic acid complementary sites should be capable of cross-reacting with the closed ring product and prostaglandins, as outlined in Figure 1.

### 2.2. Successful Imprinting in MIP

**Figure 2:**
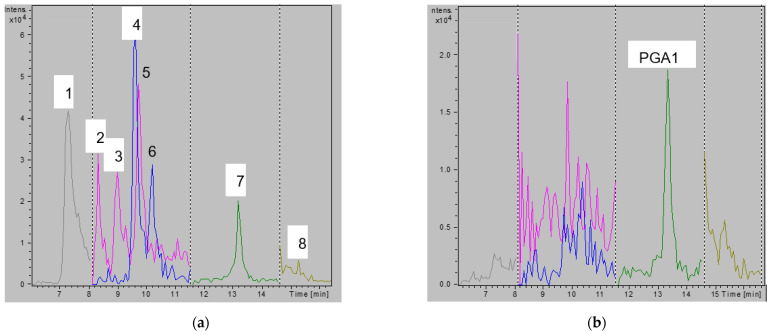
MRM chromatogram of reconstituted fractions after loading a PG mixture on MIP and after washing with CHCl_3_ (**a**) and DCM (**b**). While in (**a**) peaks corresponding to eluted compounds are present at significant intensity values, in (**b**) the same peaks are either not present or are showing a much lower intensity. Peaks were labelled as 1 = 6kPGF_1a_; 2 = 8iPGF_2a_; 3 = PGF_2a_; 4 = PGE_2_; 5 = PGE_1_; 6 = PGD_2_; 7 = PGA_1_; 8 = 15dPGJ_2_. Colors indicate the different MRM transitions monitored.

Arachidonic acid was imprinted in its free acid form, hypothesizing the dominant monomer–template interaction consisting of a donor–acceptor cyclic hydrogen-bonded motif with methacrylamide, as shown in Figure 1. In this case, MIP capture of lipid mediators from serum or plasma would hence benefit from an adjustment to acidic pH. We then investigated the ability of the newly synthesized sorbents to enrich a mixture of prostaglandin standards from acidic buffers. As shown in Figure 2, the PGs were trapped on the MIP cartridges when dissolved in 15% EtOH/1% FA. Very similar results were obtained when using NIP (data not shown). While the PGs were eluted from both sorbents when washing with CHCl_3_ (Figure 2a), they were retained when using DCM (Figure 2b). Thus, DCM was selected as a suitable washing solvent to decrease unspecific binding. While we can’t fully explain the reason for this observation, we hypothesize that the lower polarity index of DCM might affect the interaction of the analytes.

To optimize the washing procedure, we tested the effect of the addition of MeOH to the DCM solution. Since small traces of water could interfere with the hydrogen bonding interaction, all solvents were dried prior to analysis. When using only 1% MeOH, the PGs were partially eluted from the sorbent (this effect was mainly present with the NIP), as shown in Figure S5.

**Figure 3:**
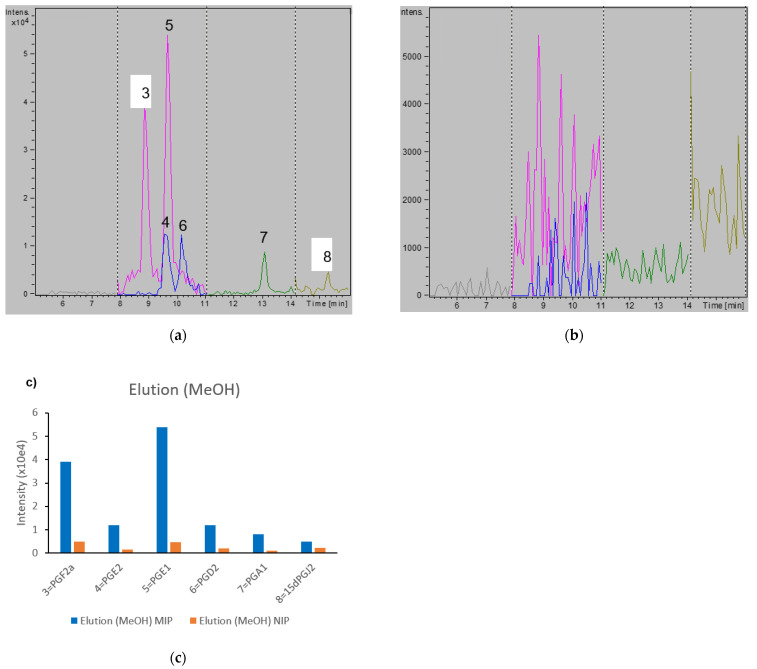
Chromatogram of reconstituted Elution fractions (MeOH) from the experiment in Figure S6. MIP elution fraction (**a**), NIP elution fraction (**b**), and corresponding peak intensities of key PGs (**c**). Numbering of PG peaks as in Figure 2. Colors indicate the different MRM transitions monitored.

When using 5% MeOH, larger differences between MIP and NIP were measured, indicating higher specificity of binding to the MIP material. Whereas PGs could be detected in the MIP wash fraction, the peak intensities were almost 10 times lower than those detected in the NIP wash fraction (Figure S6). This was confirmed by chromatograms of the eluted fractions (Figure 3), showing PGs eluting only from the MIP. Collectively, these experiments prove that imprinting was successful in enhancing affinity for the PG class of lipid mediators. All PGs were finally eluted from both the MIP and NIP when using 10% MeOH in DCM (data not shown).

### 2.3. Enrichment of Prostaglandins: Recovery Studies

**Figure 4:**
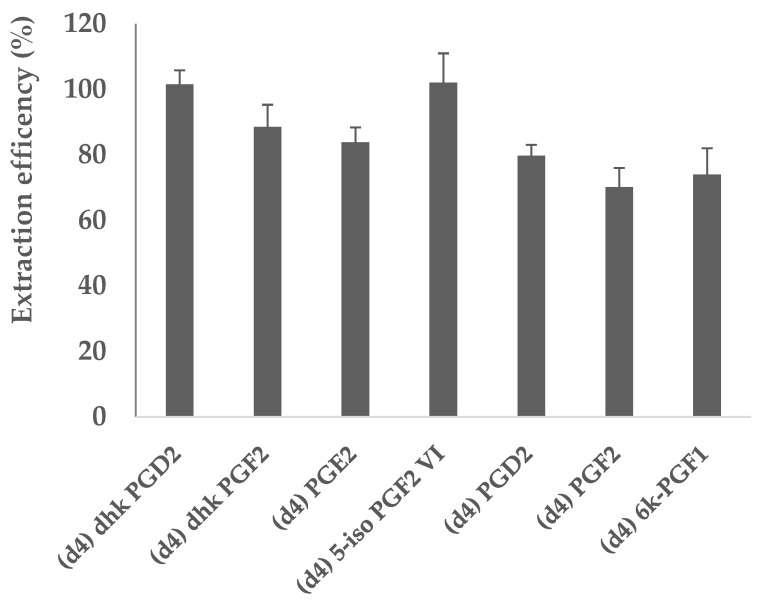
Recoveries of prostaglandin internal standards for the tested MIP-SPE protocol. Deuterated standards were added to the samples before (BE) or after extraction (AE) with MIP, and the extraction efficiency was reported as (Area peak BE/Area peak AE) × 100. The data shown represent the mean recovery percentage ±SD (*n* = 3).

As an arachidonic acid template was employed for the synthesis of MIPs and any ArA remnants within the binding cavities of the polymer could affect further analyses (memory effect), before starting any experiment involving lipid mediators, the columns were washed thoroughly with 5 mL methanol to ensure the removal of the template and the final eluent was tested for ArA presence (Figure S7). After this, we tested the recovery of prostaglandin standards from the MIP. When using the newly developed protocol, the recovery percentage of the seven prostaglandins internal standards (IS) is shown in Figure 4. The results show a reproducible recovery of all deuterated IS between 68% and 102%, comparable to the performances of the commonly used and commercially available Strata-X SPE (Figure S8).

### 2.4. Enrichment of Lipid Mediators: Optimization of the SPE Procedure 

The optimization of the SPE procedure was carried out based on two different performance criteria: (1) to achieve maximum selectivity for template structural analogs and (2) to achieve maximum recovery for the entire oxylipin class, with reference to the established Strata-X SPE procedure. The conditions evaluated during the enrichment procedure included the volume of the washing solvents, their composition, and their pH, as described below. Other aspects of the overall procedure (equilibration times, type of plastic tubes, etc.) were the same for all the different materials tested in order to increase comparability.

The main aim of the washing step in the SPE process is to reduce the adsorption of interfering substances and to limit unspecific binding, increasing the recovery of the target analytes. As the main interfering compounds in the lipid mediators analysis have been identified as relatively water-soluble molecules [30], different concentrations of methanol–water mixtures (5, 15%, and 25% methanol) were used to wash the polymer and correlated to the amount of recovered oxylipin standards spiked in commercial human plasma. The highest recovery was achieved when 15% methanol was used as washing solvent (Figure S9), and this solvent composition was used for all the subsequent experiments. The effect of the volume of washing solution (3 mL and 6 mL) was also investigated. The results show that no difference in the recovered lipid mediators amount was detected after washing with different volumes (Figure S10); thus, 3 mL became the volume of choice. 

As lipid mediators have hydroxyl groups that may be present in different ionization states, we tested the influence of pH on the enrichment process. The addition of a variable percentage of acetic acid (0.1–1%) to the loading and washing solvents was used to favor binding between analytes and the MIP, possibly enhancing the enrichment factor [31]. The highest recoveries were obtained with 0.5% acetic acid, although the difference was significant only for few specific cases (Figure S11). Therefore, 0.5% acetic acid was used for all the extraction processes.

**Figure 5:**
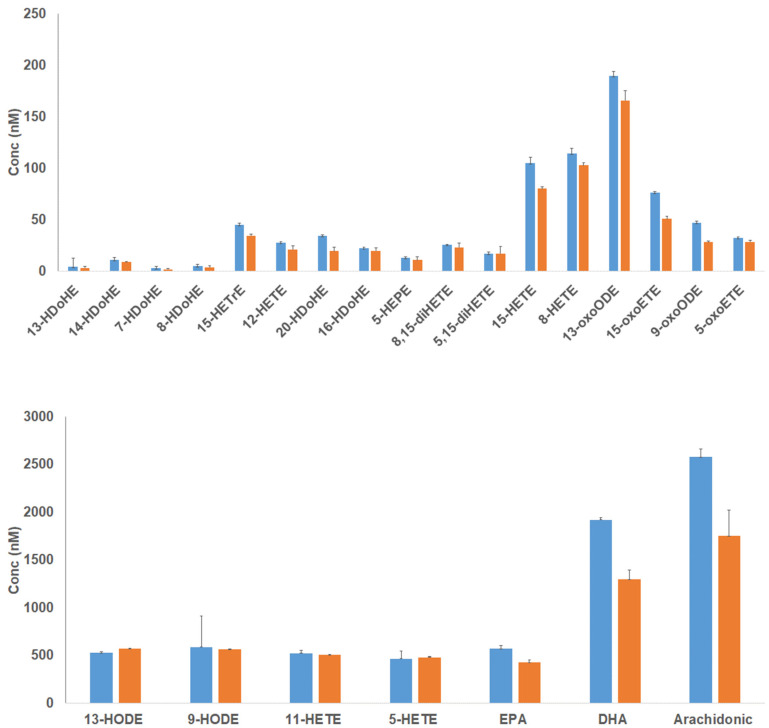
Comparison between the use of MIP (blue) and NIP (orange) polymers for blood plasma lipid mediators enrichment when using 0.5% acetic acid in the washing solvent. The same plasma extracts were used to test both materials. Bar charts represent Mean ±SD (*n* = 3). SD—standard deviation.

In agreement with the synthetic standard extractions in Figure S6, significantly higher recoveries were observed using the MIP sorbent but, importantly, not for all compounds tested. A closer inspection revealed a MIP higher affinity for templates containing long-chain polyunsaturated fatty acids, notably 20-HDoHE, arachidonic acid, and DHA. Moreover, given the reduced preference of MIP for, e.g., 15-oxoETE and 15-HETE, we hypothesize that a polar substituent closer to the carboxylic acid head group might reduce the binding to the imprinted sites (Figure 5). 

**Figure 6:**
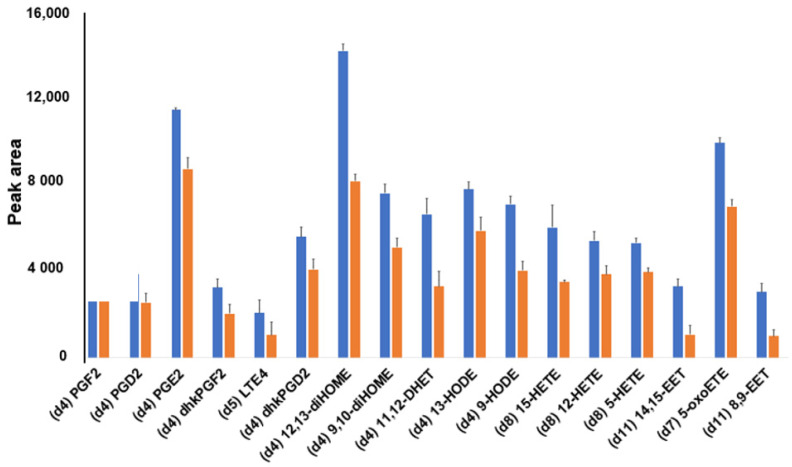
Comparison between MIP (blue) and NIP (orange) polymers for lipid mediators enrichment with 15% methanol and 0.5% acetic acid in the washing solvent. A mixture of internal standards spiked into blood plasma was used to test the recovery affinity of both materials. Bar charts represent Mean ±SD (*n* = 3). SD—standard deviation.

Similar enhanced imprinting effects were observed upon optimizing the amount of methanol in the washing solvent. Using 15% methanol (Figure 6) led to a higher affinity between MIP and open-chain template-analogous structures (e.g., (d4)12,13-diHOME, (d11)11,12 DHET, and (d4)9-HODE), in addition to a weak preference for prostaglandins, in agreement with the results obtained under different conditions and shown in Figure 3.

In order to reach the highest recovery of lipid mediators following the optimization criteria described above, different conditions for the elution (90% chloroform +10% methanol vs. 100% methanol) were tested and compared. Two consecutive elutions of 500 µL each were performed, and no significant differences between the two solvent systems were found (Figure S12). Therefore, we chose the less toxic and more easily disposable methanol as the only solvent for the elution step.

### 2.5. Comparison between MIP/NIP and Strata-X for the SPE-Based Enrichment of Lipid Mediators

#### 2.5.1. Enrichment of Lipids in Human Plasma

**Figure 7:**
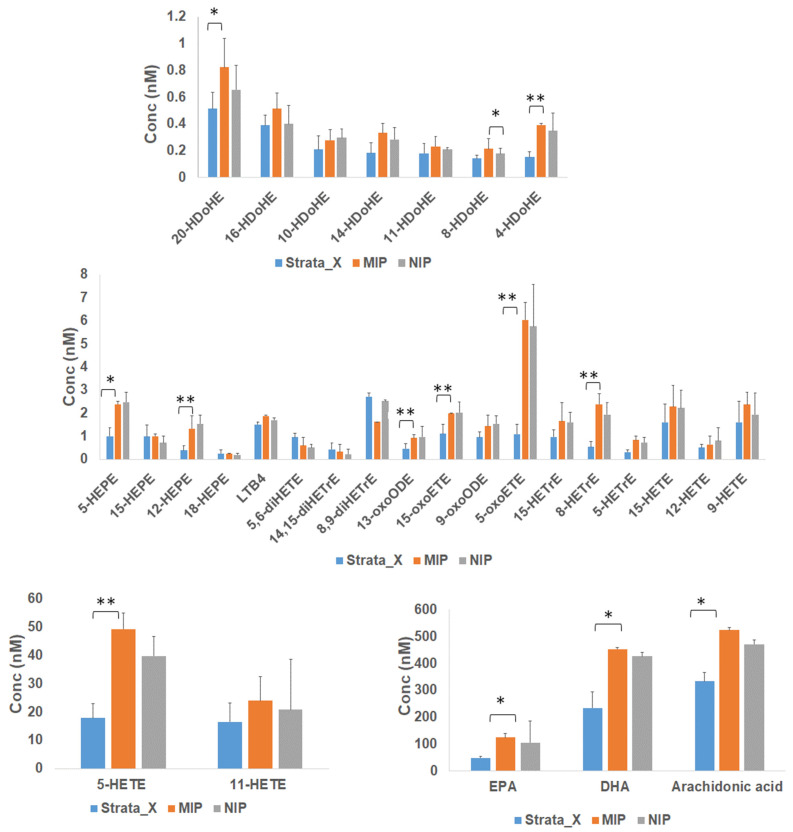
Comparison between MIP and NIP polymers and the commercially available Strata-X for lipid mediators enrichment from human plasma extracts. The same plasma extracts were used to test all the materials. Bar charts represent Mean ±SD from *n* = 3. SD—standard deviation. Stars represent significant differences calculated by Student’s *t*-test. * Indicates significant changes with a *p* value < 0.05. ** Indicates significant changes with a *p* value < 0.01.

The aim of this work was to explore the use of newly synthesized polymers for the enrichment of biologically relevant lipid mediators. Currently, the use of SPE-based methods for the extraction of lipid mediators from biological samples is a common procedure due to their low concentrations, and it is used to decrease the matrix effect in complex samples and to concentrate the target molecules. The most popular experimental protocols for the analysis of lipid mediators by LC–MSMS include the use of SPE Strata-X cartridges (8B-S100-UBJ, Phenomenex) at the sample preparation stage [7,32]. Thus, we compared the performances of our newly synthesized MIP/NIP polymers to the Strata-X cartridge for the extraction of endogenous lipid mediators from biological samples. The enrichment of lipid mediators from 20 µL of commercial human plasma was carried out with the optimized protocols described in the previous paragraphs (Figure S4) and in Section 2.4. As it can be seen from Figure 7, MIP/NIP and Strata-X sorbents gave comparable results.

While optimization with the goal of maximizing recovery could compromise the selectivity of the polymers towards specific classes of lipid mediators, the results in Figure 7 showed a slightly higher affinity of MIP for HDoHEs, HEPEs, HETEs, oxo-EETs, HETrEs, and PUFAs when compared to NIP and Strata-X. However, the Strata-X cartridges showed a higher yield than MIP and NIP for diHETrEs (Figure 7 with representative chromatograms showing the different yields for four species are shown in Figure S13). These results confirmed that these new sorbents are suitable for the extraction of low levels of lipid mediators from biological samples and that their performance is comparable to routinely used procedures (Table S1). We would also like to highlight that our newly synthesized polymers are stable over a long time and that the same material can be used multiple times after performing proper washing steps, increasing its cost-effectiveness.

#### 2.5.2. Enrichment of Lipids from Mouse Tissues

As an additional application, we tested these new materials for the extraction of lipid mediators from murine tissues. The optimized SPE-LC–MS/MS method was applied to a pooled sample of muscle tissue from wild type mice. Samples were prepared as reported in the methods section. Lipid mediators were extracted by both MIP/NIP and Strata-X SPE systems, and the results were compared (Figure S14). In agreement with the results obtained for the human plasma lipid mediators (Figure 7), the newly synthesized polymers achieved performance comparable to Strata-X (Figure S14), with MIP showing slightly better retention than Strata-X and NIP when considering HETEs, HDoHEs, HEPEs, oxo-EETs, and DHA.

## 3. Materials and Methods

### 3.1. Reagents

All 24 deuterated lipid mediators internal standards were purchased from Cayman Chemical (Ann Arbor, MI, USA). Optima LC–MS grade acetonitrile (ACN), ethanol (EtOH), methanol (MeOH), and Isopropanol (IPA) were obtained from Fisher Scientific (Hampton, VA, USA). Acetic acid was obtained from Baker A.C.S. Reagent (Radnor, PA, USA).

Stearic acid (SA) was obtained from Fluka (Buchs, Switzerland). Dry chloroform (CHCl_3_, ≥99%) methacrylamide (MAAM), ethylenglycol dimethacrylate (EGDMA), and azobisisobutyronitrile (AIBN) were purchased from Sigma-Aldrich (Steinheim, Germany).

### 3.2. Polymer Synthesis

Imprinted polymers were prepared in the following manner. Arachidonic acid (ArA) (0.2 mmol), MAAM (4 mmol), and EGDMA (20 mmol) were dissolved in dry chloroform (5.6 mL). The initiator AIBN (1% *w/w* of total monomers, 43.65 mg) was added to the solution, which was transferred to a glass ampoule, cooled to 0 °C and purged with a flow of dry nitrogen for 10 min. The tubes were then flame-sealed while still under cooling, and the polymerization was initiated by placing the tubes on a roller at 10 °C in a thermostatted photoreaction chamber followed by UV irradiation. The latter was performed using a high-pressure Hg vapor lamp (Philips, HPK 125 W) immersed in a thermostatted water bath and placed at 5 cm from the polymerization vessel. The tubes were then broken and the polymers lightly crushed and extracted in a Soxhlet apparatus with methanol for 24 h. This was followed by further crushing and sieving, whereby the fractions 25–36 µm and <25 µm were used for packing the solid-phase extraction cartridges or filter plates. Non-imprinted polymers (P_N_#) or stearic acid imprinted polymers were prepared in the same manner as described above, but with the omission or replacement of the template molecule. The collected particles of size <25 μm were subsequently fractionated by repeated sedimentation as described before (Figure S2) [33].

Glass vials (20 mL, Assistent, Sondheim, Germany) containing the particles were filled with MeOH/H_2_O (grade 2 water) (80/20, *v/v*). The suspension was shaken and allowed to sediment for ca. 30 min, followed by removal of the supernatant. This procedure was repeated until the supernatant was clear. The sediments were dried in an oven overnight at 50–60 °C.

### 3.3. Preliminary SPE Experiments Using Synthetic Prostaglandins

The offline testing of the MIPs and NIPs was done using packed 1.5 mL solid-phase extraction (SPE) cartridges (Agilent Technologies, Palo Alto, CA, USA). About 30 mg of packing material (d_p_ 25–36 µm) were added 300 µL MeOH, and the slurry was treated with ultrasound for 15 min. Then, the slurry was vacuum aspired into an empty SPE cartridge. The cartridges were conditioned using 2 mL MeOH followed by 2 mL H_2_O. Prostaglandins were solved in 15% EtOH/1% formic acid. The samples were percolated through the cartridge, and the cartridge was dried for 10 min. Then, washing was performed using either CHCl_3_, dichloromethane (DCM), or DCM containing x% MeOH, followed by drying for another 10 min. Elution was performed using MeOH. Both the washing solvents and the elution solvents were collected; the solvent was evaporated under a gentle stream of nitrogen (99.996%, AGA, Oslo, Norway), re-dissolved in 30% EtOH/1% FA, filtered, and injected onto a capillary liquid chromatographic (cLC) system with tandem mass spectrometric (MS/MS) detection as reported elsewhere [17].

### 3.4. SPE Procedure for Analyzing Lipid Mediators

The polymer particles were manually packed into Captiva 0.2 µm polypropylene 96-well 1 mL filter plates (Agilent technologies, Santa Clara, CA, USA), equipped with a polyethylene frit disk (20-μm pore size) as a plug at the bottom of the well and a soft membrane at the top (Figure S3). Twenty milligrams of each polymer were initially conditioned in 4 mL methanol (MeOH) and pipetted into each well, allowing the solvent to flow through. The polymers were pulled down further by adding 1 mL of methanol again. Three different cartridges were then used for the MIP and for the NIP.

Aliquots of 50 µL commercially available plasma (Pooled Healthy Human Plasma, Seralab, Sussex, UK) were thawed at room temperature. Specific SPE protocols were used for MIP, NIP, and Strata-X cartridges (8B-S100-UBJ, Phenomenex) (Figure S4I & II).

The use of the Strata-X for eicosanoid extraction was performed according to an earlier method with some modifications [26,34,35]. After enrichment, the eluted compounds were dried completely using a SpeedVac concentrator. The extracts were reconstituted in 50 μL of ACN/water/acetic acid (60/40/0.02, *v/v*). The samples were injected and analyzed by UHPLC/MS/MS.

For the analysis of wild-type mouse (C57BL6) tissue samples, the tissues were homogenized in 100% methanol after lyophilization. The lipid mediators are extracted from homogenates by SPE following the identical purification protocol as for the plasma samples. All animal procedures were performed in accordance with the Guidelines for Care and Use of Laboratory Animals of the University of Basel and approved by the Animal Ethics Committee of Kantonales Veterinäramt of the Kanton Basel-Stadt, permit n. 2175, 26-1-2012.

### 3.5. Liquid Chromatography–Mass Spectrometry (LCMS) Procedure for Analyzing Lipid Mediators

The LCMS lipid mediators analysis was performed based on previous publications [26,36]. Briefly, high-performance liquid chromatography (HPLC) was performed using an Agilent 1290 series chromatographer (Agilent, Santa Clara, CA, USA). Reversed-phase separation was achieved by Acquity UPLC BEH shield RP18 column (2.1 × 100 mm; 1.7 m; Waters) and maintained at 40 °C. The mobile phase was a gradient of solvents A (ACN/water/acetic acid (60/40/0.02, *v/v*)) and B (ACN/IPA (50/50, *v/v*) with a flow rate of 0.5 mL/min. The stepwise gradient conditions were carried out for 10 min as follows: 0–5.0 min, 1–55% of solvent B; 5.0–5.5 min, 55–99% of solvent B, and finally 5.5–6.0 min, 99% of solvent B. Injection volume was 10 μL, and all samples were kept at 4 °C throughout the analysis.

The HPLC system was coupled to Agilent 6495 triple-quad mass spectrometer (Agilent, Santa Clara, CA, USA). The electrospray ionization was conducted in negative mode, and the dynamic MRM option was used and performed for all compounds with optimized transitions and collision energies. The determination and integration of all peaks were manually performed using the Mass Hunter Workstation software (Agilent, Santa Clara, CA, USA).

### 3.6. Statistical Analysis

The data were mostly analyzed using GraphPad Prism version 6 software (GraphPad Software, La Jolla, CA, USA) for Windows and partially using Microsoft Excel version 2013.

## 4. Conclusions

Recently, particular attention has been directed towards the development of MIPs as selective materials for extracting several small metabolites, including lipids [37]. Our groups particularly focused on the use of these new materials for the enrichment of bioactive lipids that are present at low concentrations in biological samples [38]. This work demonstrates the applicability of new MIP/NIP-based SPE for the selective extraction of lipid mediators from human plasma and mouse tissues. Depending on the SPE conditions, selectivity can be tuned to be either narrow, with compromised overall recovery, or broad. In the former case, the MIP reveals a receptor-like behavior, with preferred retention for structural analogs of the template. This is of interest for the future development of targeted extractions of specific PG biomarkers [39]. In contrast, the latter conditions offer an alternative procedure to the more established comprehensive profile of lipid mediators. This method is based on an SPE enrichment step using MIP/NIP cartridges followed by UPLC–MS/MS analysis. Overall, our data demonstrate that the MIP/NIP-SPE is at least comparable to the most popular and commercially available Strata-X. In terms of structural specificity, MIP accomplished better retention of PGs, HDoHE, HEPE, HETE, oxo-EET, HETrE, and PUFA compounds, while the Strata-X recovery was higher for diHETrEs.

To the best of our knowledge, this is the first time that MIP and NIP have been shown to be selective for lipid mediators from plasma and tissue samples. This fundamentally selective retention behavior gives relevance to the future employment of MIP/NIP in biological sample cleanup, with special attention towards more cost-effective analyses of lipid mediators. This work, together with others, expands the use of imprinted polymers in lipidomics for large scale studies and possible clinical research application, as the target molecules are involved in important physiological processes.

## Data Availability

All data are available as supplementary material or could be retrieved by contacting the corresponding authors.

## Supplementary Materials

The following are available online at https://www.mdpi.com/article/10.3390/metabo11080539/s1, Figure S1: Representative chemical structures of lipid mediators (DHA, 20-HETE, 20-HDoHE, PGE2) and general procedure for their off-line solid phase extraction, Figure S2: The polymer particles subsequently fractionated by repeated sedimentation, Figure S3: The schematic shows the packing of polymer into the cartridge used for the reported experiments, Figure S4: Schematic representation of the two distinct solid-phase extraction procedures performed on two separate MIP and NIP-SPE (I) and Strata-X (II) extraction cartridges, Figure S5: Chromatograms of reconstituted wash fractions after washing with 1% MeOH in DCM from MIP (a) and NIP (b), Figure S6: Chromatogram of reconstituted Wash fractions after washing with 5% MeOH in DCM from MIP (a) and NIP (b) and corresponding peak intensities of key PGs (c), Figure S7: Arachidonic acid template (ArA) was used for the synthesis of MIP. After washing the column with 5 mL the template was almost completely removed, Figure S8: Extraction recovery of deuterated standards using Strata-X SPE, Figure S9: Bar charts showing the effect of the washing solvent composition on the recovery of lipid mediators using MIP polymer, Figure S10: Selection of appropriate volume of washing solvent (3 mL and 6 mL). The data shows that there is no difference in the recovery when the volume of washing solvent increases from 3 mL to 6 mL, Figure S11: Optimization of acidity (0.1%, 0.5% and 1% acetic acid) in the Washing solvent composition, Figure S12: Selection of appropriate elution solvent conditions (90% chloroform (CHCl3): 10% Methanol and 100% Methanol). The data shows that there is no significant difference in the recovery between the two conditions, Figure S13: Representative chromatograms showing 16-HDoHE (A), 15-oxoETE (B), 12-HETE (C) and DHA (D) enriched with MIP, NIP and Strata-X, Figure S14: Comparison between the lipid mediators binding properties of MIP/NIP polymers and commercially available Strata-X when using murine muscle tissue extracts, Table S1: Comparison between the analyte levels obtained with the use of our approach and the ones reported previously from the same type of biological matrix.
