# Supplementary material for: Tailored Polymer-Based Selective Extraction of Lipid Mediators from Biological Samples
Source: Metabolites. 2021 Aug 13;11(8):539. doi: 10.3390/metabo11080539 (PMC8398397; doi:10.3390/metabo11080539)
Supplement: Supplementary file 1 [file metabolites-11-00539-s001.zip › metabolites-1276203-SI.pdf]

Supplementary materials

Title: Tailored polymer-based selective extraction of oxylipins from biological samples

Authors:
Yohannes Abere Ambaw[1,2,3], Sandra Rinne Dahl[4,5], Yan Chen[4], Tyge Greibrokk[4], Elsa Lundanes[4], Issam Lazraq[6], Sudhirkumar Shinde[6], Jayashree Selvalatchmanan[1,2], Markus R Wenk[1,2], Börje Sellergren[6*] and Federico Torta [1,2*]

Affiliations:
[1] Department of Biochemistry, Yong Loo Lin School of Medicine, National University of Singapore, [2] SLING, Singapore Lipidomics Incubator, Life Sciences Institute, National University of Singapore, Singapore, [3]Department of Molecular Metabolism, Harvard T.H.Chan School of Public Health, Harvard University, USA, [4] Department of Chemistry, University of Oslo, Norway, [5] Hormone Laboratory, Department of Medical Biochemistry, Oslo University Hospital, Norway, [6]Department of Biomedical Sciences, Biofilms Research Center for Biointerfaces, Faculty of Health and Society, Malmö University, Sweden

	**This study, MIP (nM)**	***Chen et al* (nM)**
20-HDoHE	9.38 ± 0.01	8.59 ± 0.63
16-HDoHE	5.83 ±1.27	7.94 ± 0.08
10-HDoHE	3.15 ± 0.23	6.13 ± 0.13
14-HDoHE	3.78 ± 0.16	6.49 ± 0.28
4-HDoHE	4.44 ± 2.02	15.22 ± 0.59
15-HEPE	11.42 ± 0.56	11.26 ± 0.05
12-HEPE	15.25 ± 2.34	11.94 ± 0.45
18-HEPE	2.96 ± 0.08	2.62 ± 0.23
13-oxoODE	10.64 ± 1.62	6.53 ± 0.45
15-oxoETE	22.51 ± 2.01	1.79 ± 0.45
15-HETrE	18.75 ± 1.40	14.30 ± 0.04
15-HETE	25.88 ± 0.34	32.17 ± 2.05
12-HETE	7.33 ± 0.34	11.59 ± 0.42
5-HETE	561.21±15.70	254.18 ± 1.31
11-HETE	272.24 ±12.47	130.14 ± 1.29
Arachidonic acid	5979.25 ± 559.94	3855.78 ± 52.70

Table S1. Comparison between the analyte levels obtained with the use of our approach and the ones reported previously [Chen, G.Y. and Q. Zhang, Comprehensive analysis of oxylipins in human plasma using reversed-phase liquid chromatography-triple quadrupole mass spectrometry with heatmap-assisted selection of transitions. Anal Bioanal Chem, 2019. 411(2): p. 367-385] from the same type of biological matrix.

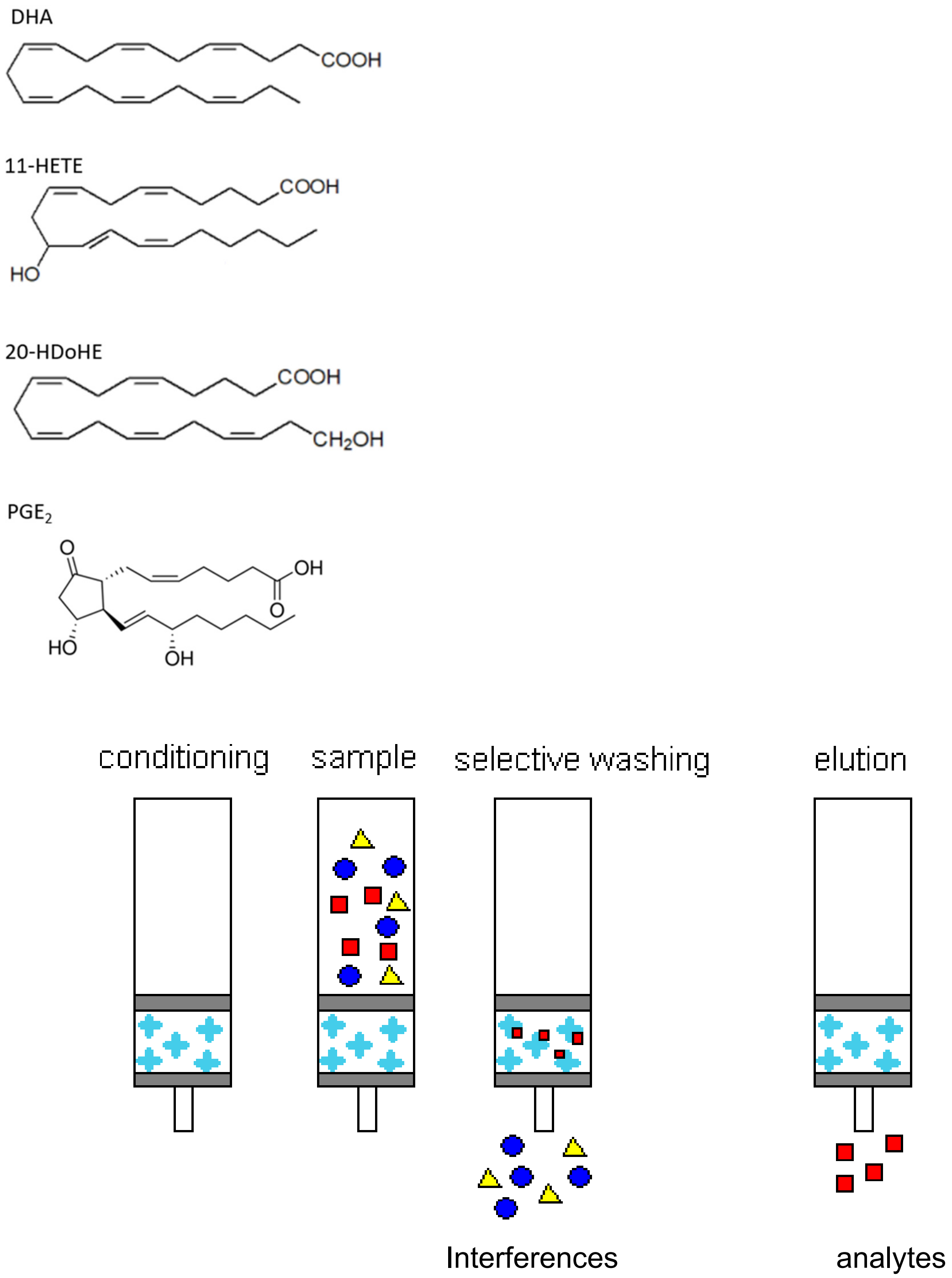

Figure S1. Representative chemical structures of oxylipins (DHA, 20-HETE, 20-HDoHE, PGE2) and general procedure for their off-line solid phase extraction.

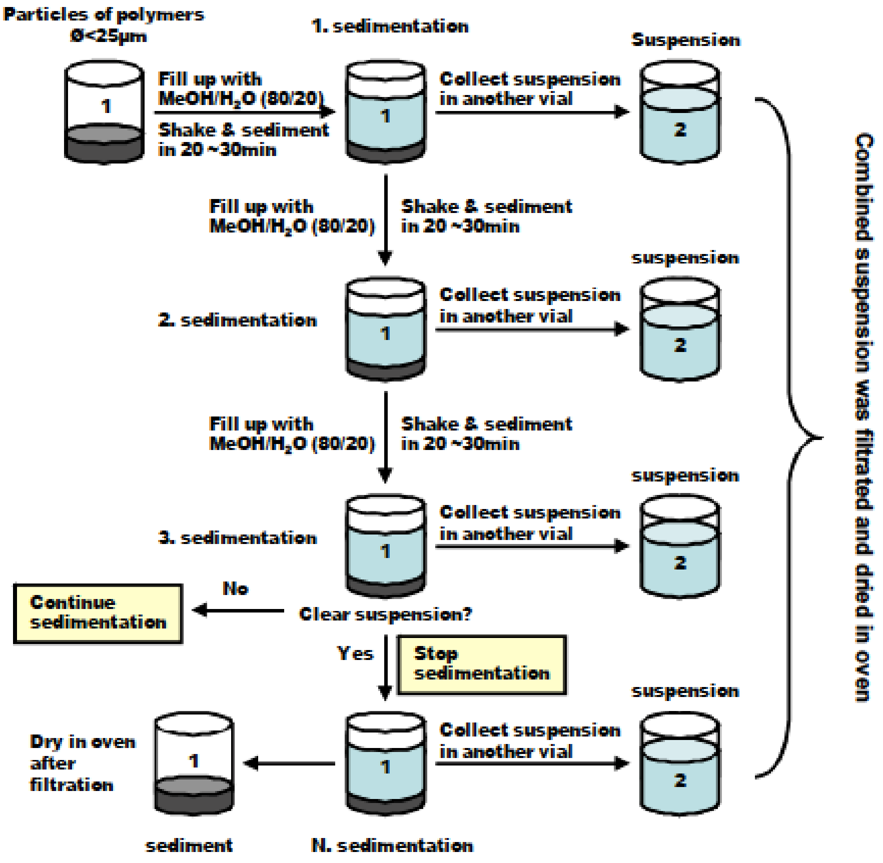

Figure S2. The polymer particles subsequently fractionated by repeated sedimentation [23].

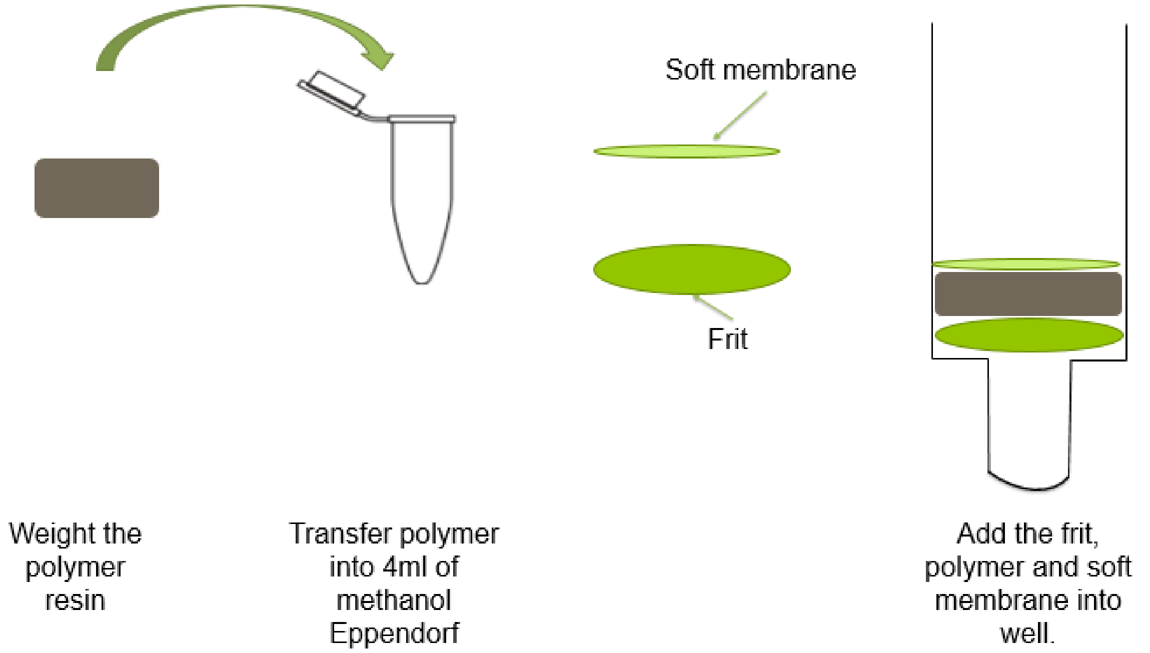

Figure S3. The schematic shows the packing of polymer into the cartridge used for the reported experiments.

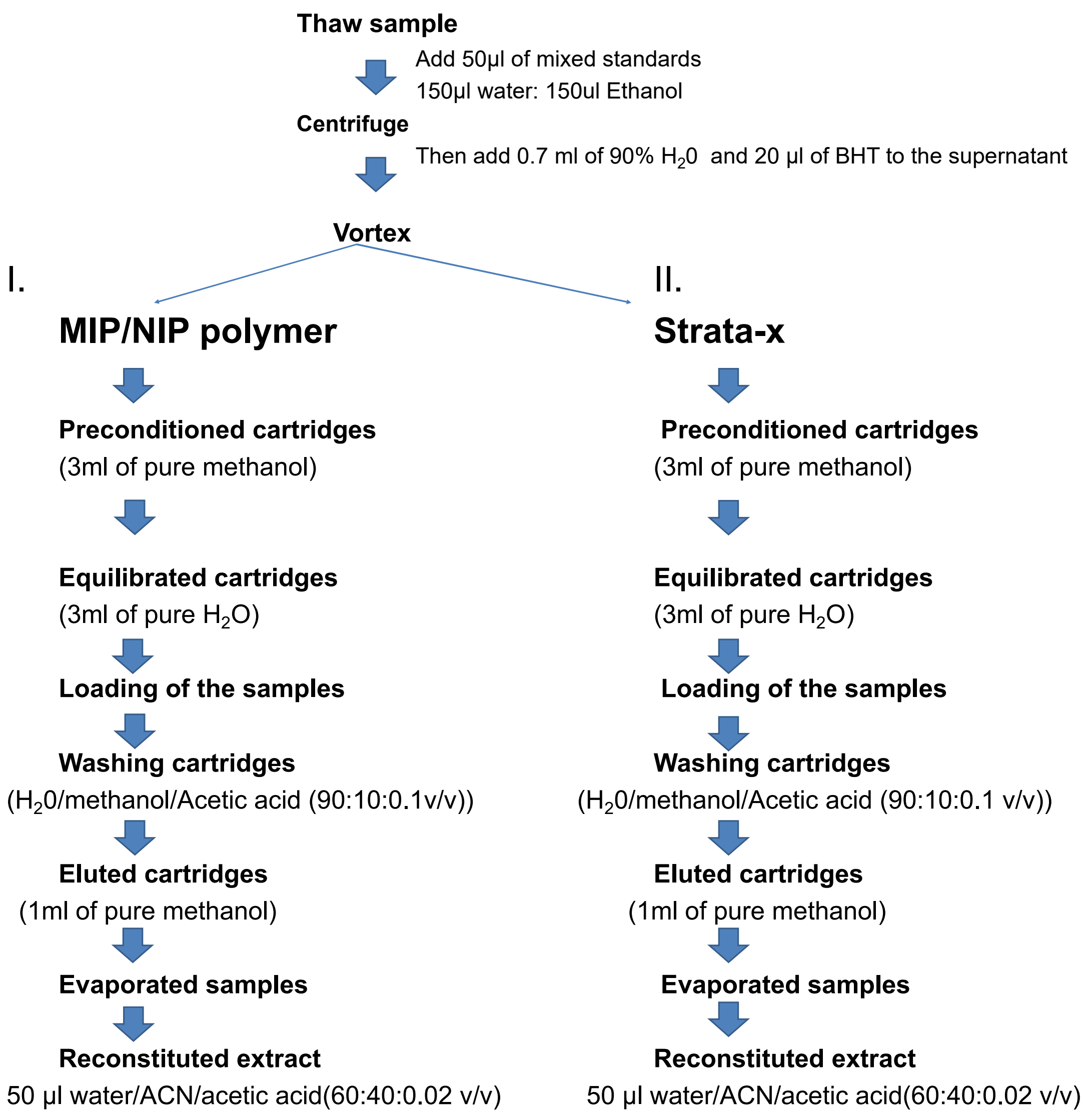

Figure S4. Schematic representation of the two distinct solid-phase extraction procedures performed on two separate MIP and NIP-SPE (I) and Strata-X (II) extraction cartridges.

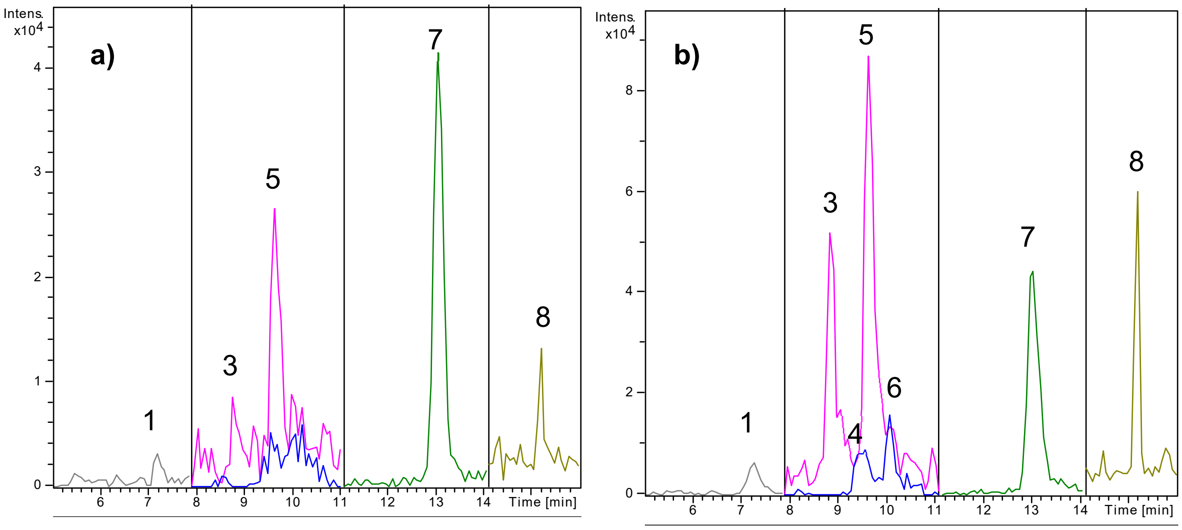

Figure S5. Chromatograms of reconstituted wash fractions after washing with 1% MeOH in DCM from MIP (a) and NIP (b). Numbering of PG-peaks was the same as in figure 2.

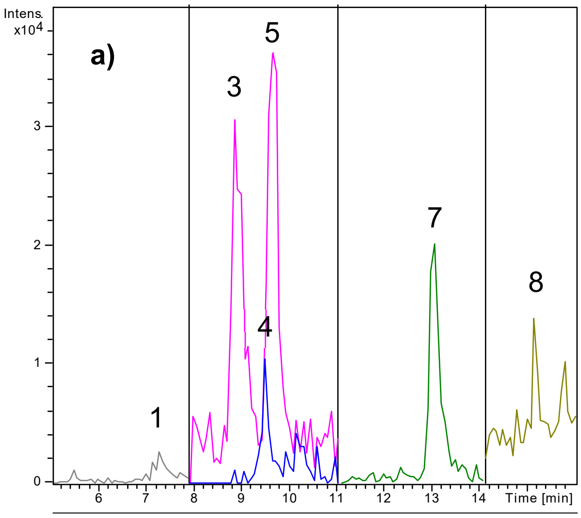

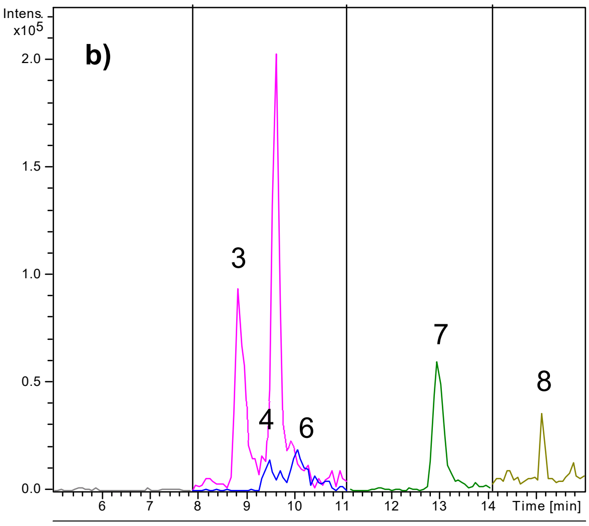

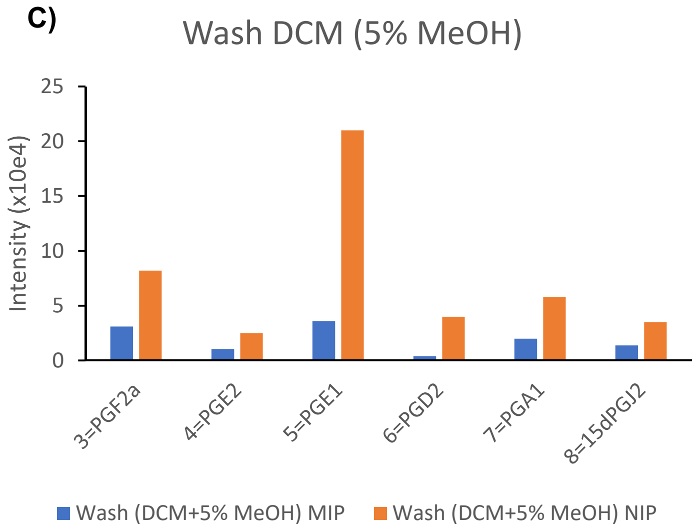

Figure S6. Chromatogram of reconstituted Wash fractions after washing with 5% MeOH in DCM from MIP (a) and NIP (b) and corresponding peak intensities of key PGs (c). Numbering of PG-peaks as in figure 2.

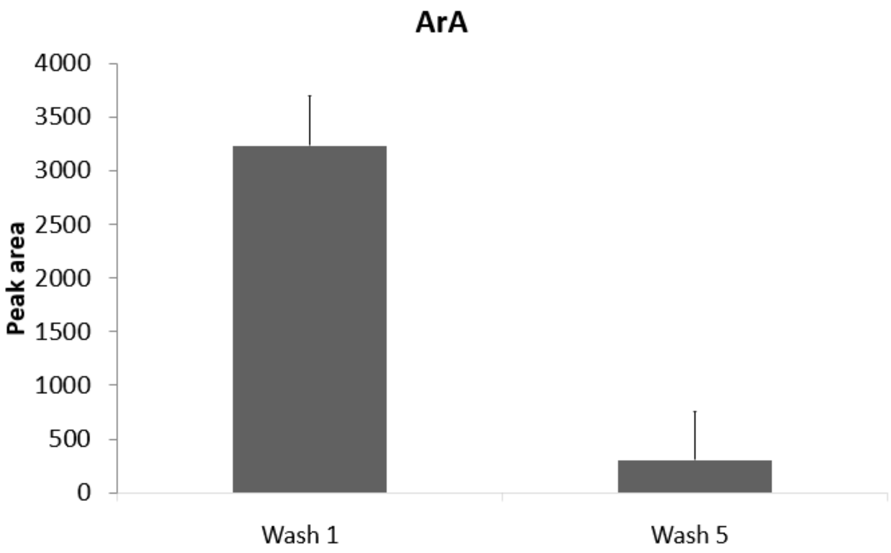

Figure S7. Arachidonic acid template (ArA) was used for the synthesis of MIP. After washing the column with 5 ml the template was almost completely removed.

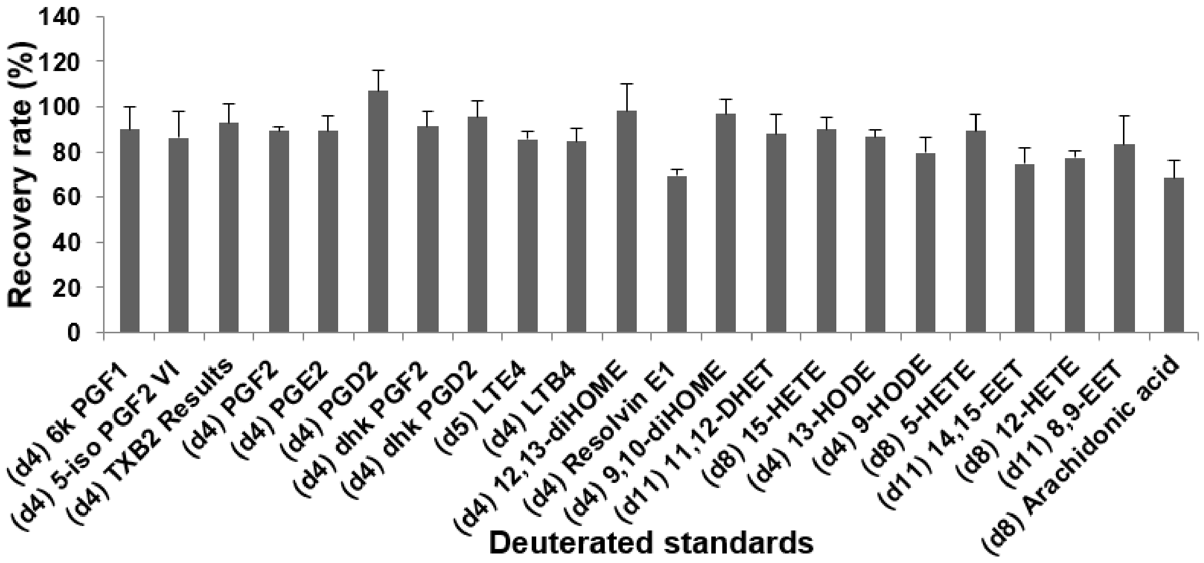

Figure S8. Extraction recovery of deuterated standards using Strata-X SPE.

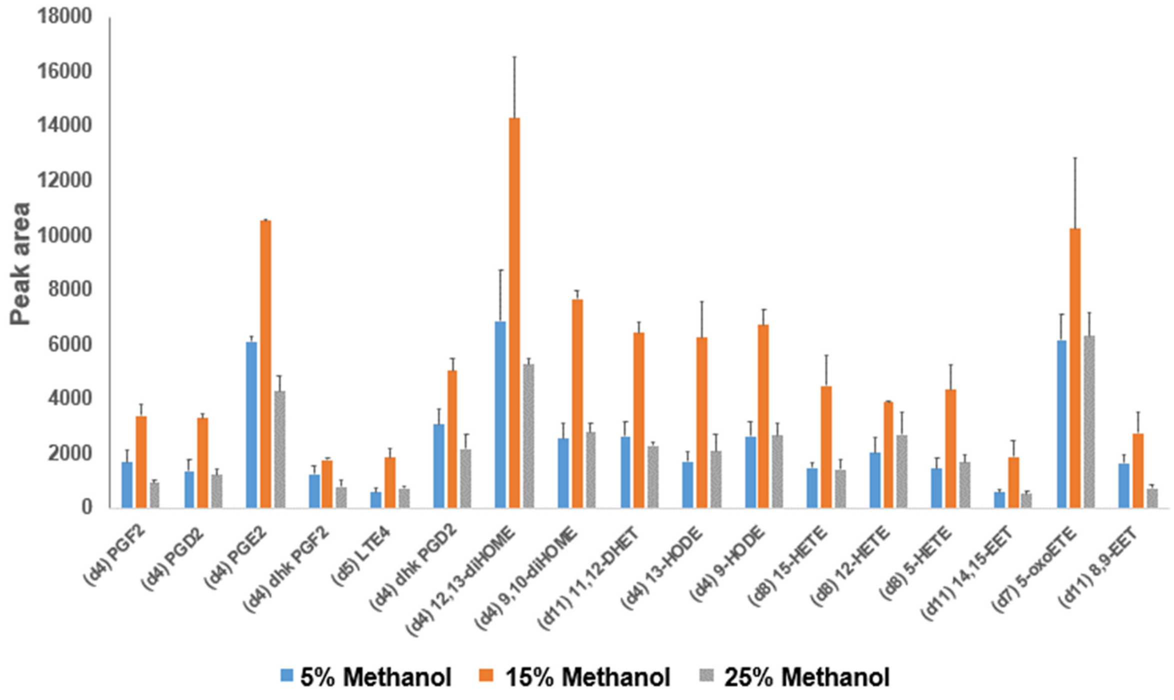

Figure S9. Bar charts showing the effect of the washing solvent composition on the recovery of oxylipins using MIP polymer. The data are presented as mean ± SD (n = 3). SD, Standard deviation.

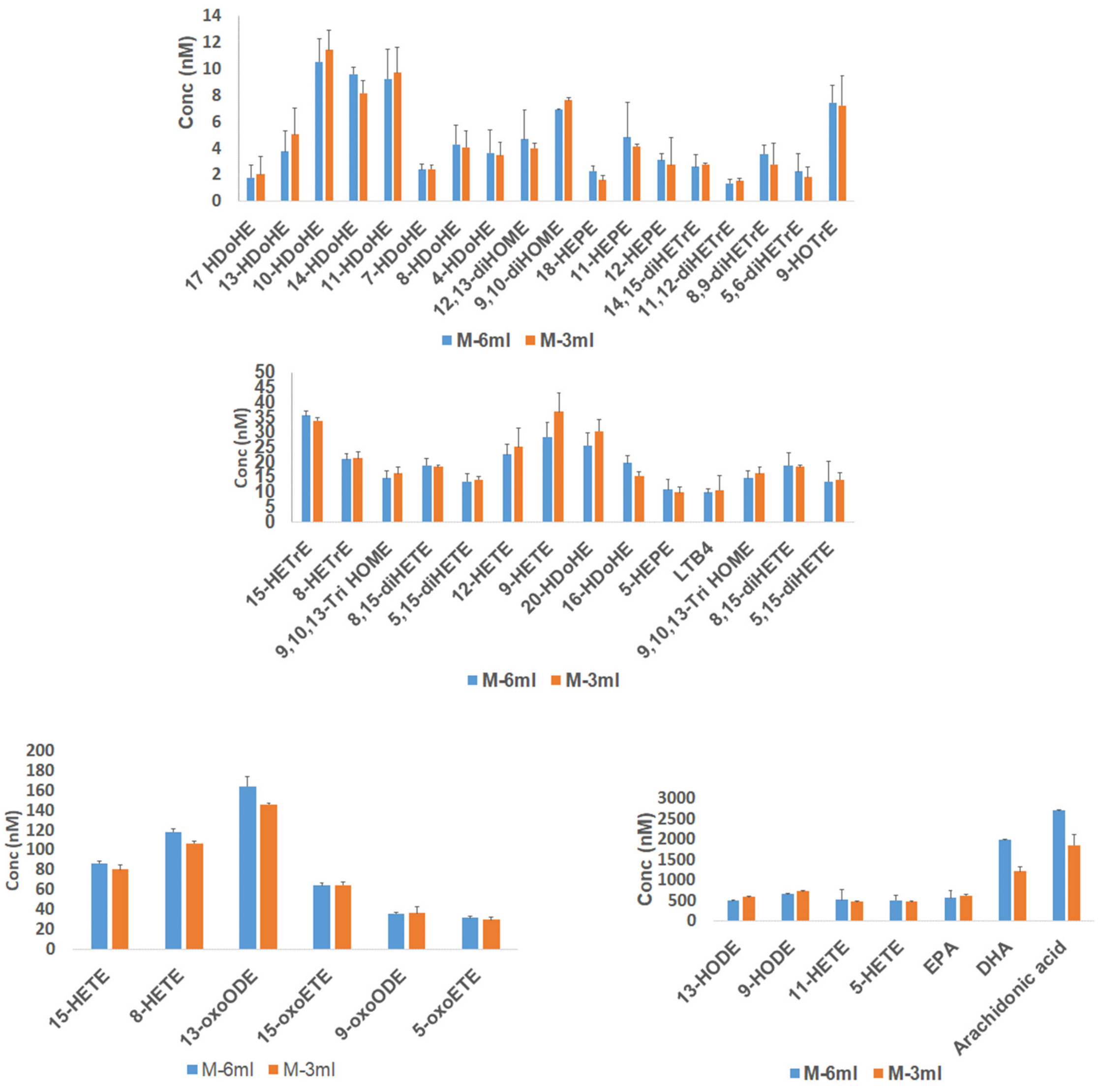

Figure S10. Selection of appropriate volume of washing solvent (3ml and 6ml). The data shows that there is no difference in the recovery when the volume of washing solvent increases from 3 ml to 6 ml. Mean ± SD were plotted. SD, Standard Deviation.

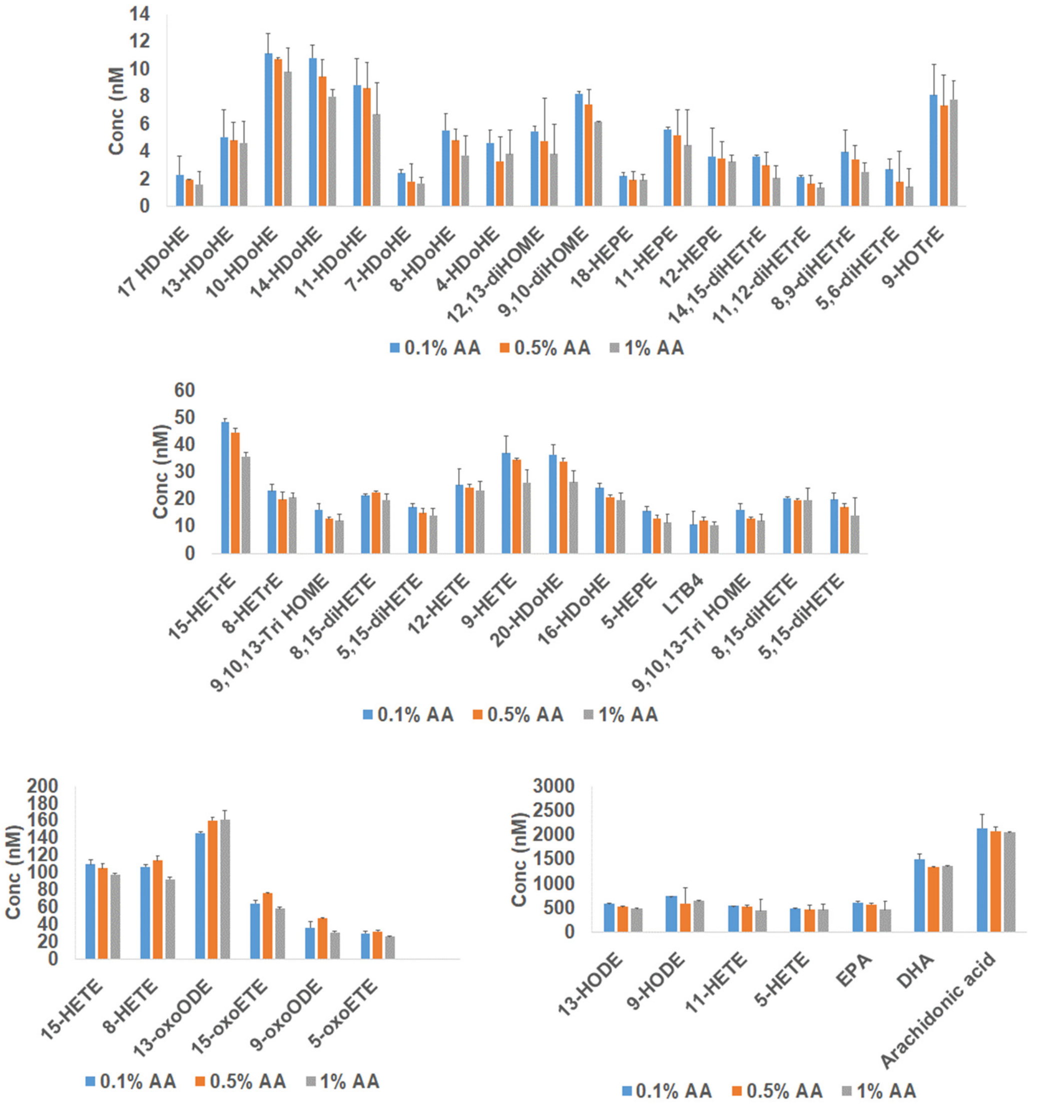

Figure S11. Optimization of acidity (0.1 %, 0.5 % and 1% acetic acid) in the Washing solvent composition. Bar charts represent Mean ± SD from n = 3. SD; Standard deviation.

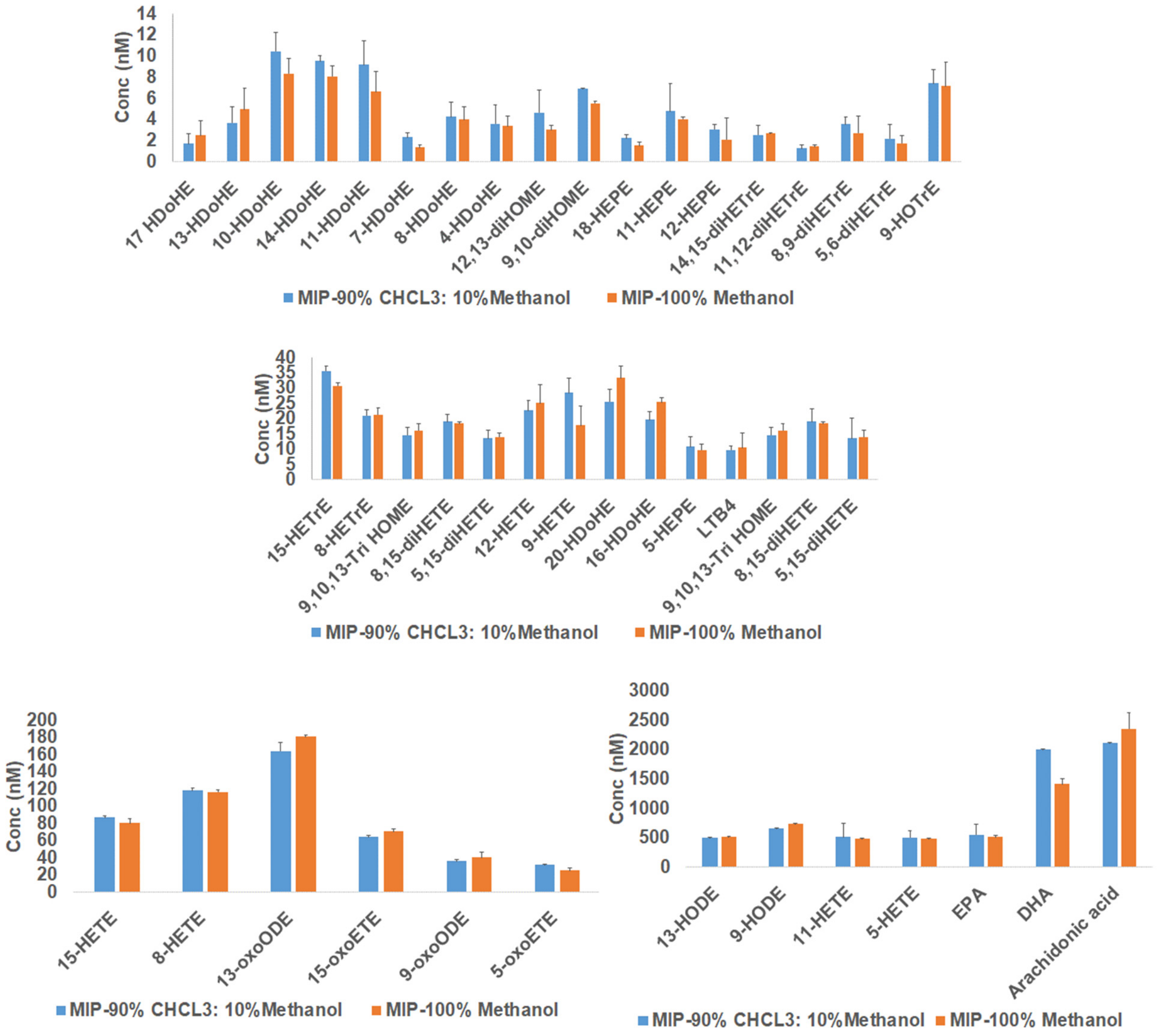

Figure S12. Selection of appropriate elution solvent conditions (90% chloroform (CHCl3):10%Methanol and 100 %Methanol). The data shows that there is no significant difference in the recovery between the two conditions. Mean ± SD were plotted. SD, Standard Deviation.

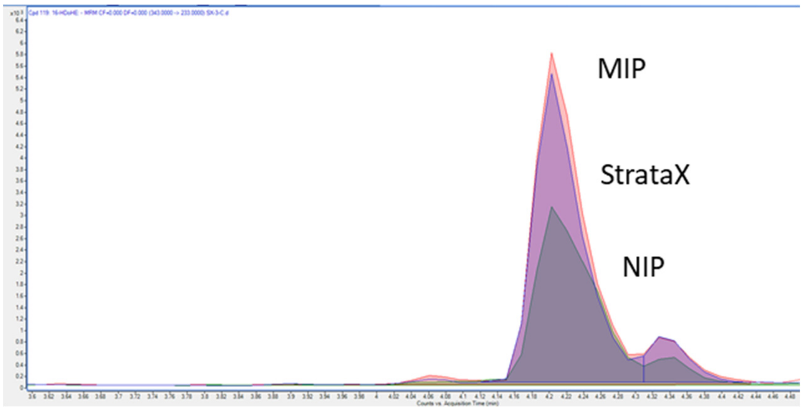
MIP
StrataX
NIP

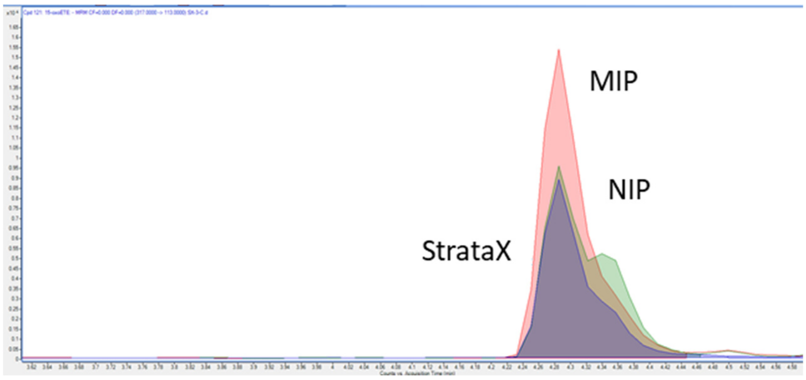
MIP
NIP
StrataX

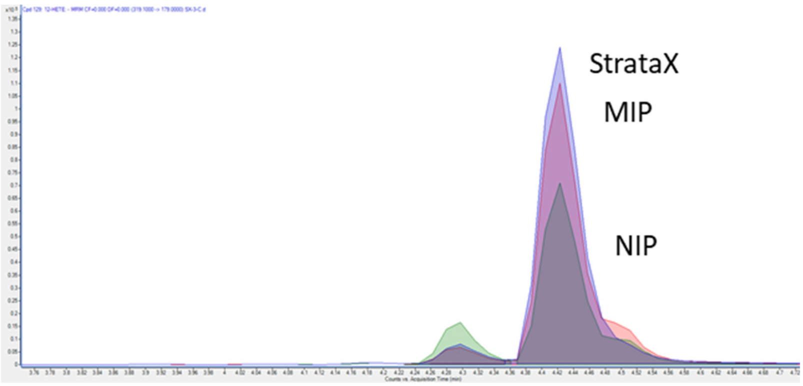
StrataX
MIP
NIP

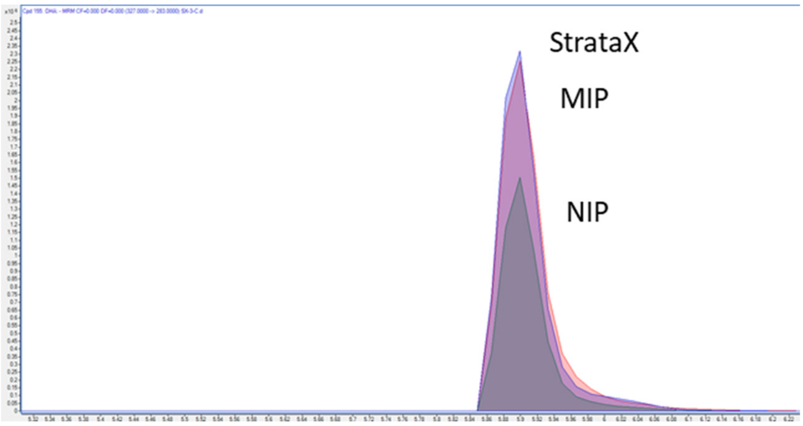
StrataX
MIP
NIP

Figure S13. Representative chromatograms showing 16-HDoHE (A), 15-oxoETE (B), 12-HETE (C) and DHA (D) enriched with MIP, NIP and Strata-X.

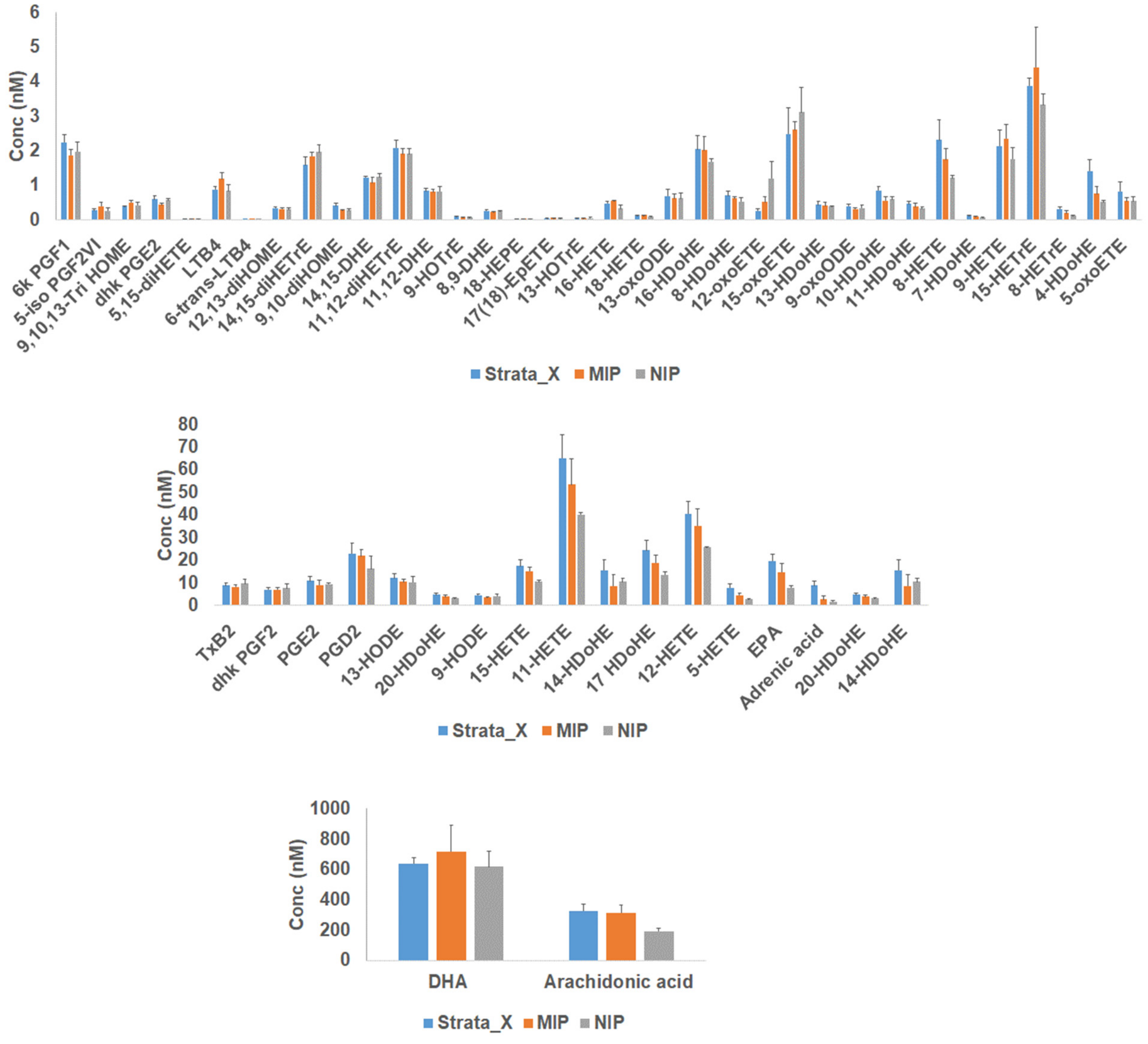

Figure S14. Comparison between the oxylipins binding properties of MIP/NIP polymers and commercially available Strata-X when using murine muscle tissue extracts. The results show that MIP have slightly better retention performances than Strata-X and NIP with PGs, HETEs, HDoHEs, HEPEs, oxo-EETs, HETrEs, and PUFAs. The same muscle extracts were used to test all the materials. Bar charts represent Mean ± SD from n = 3. SD; Standard deviation.
